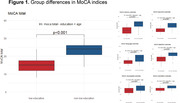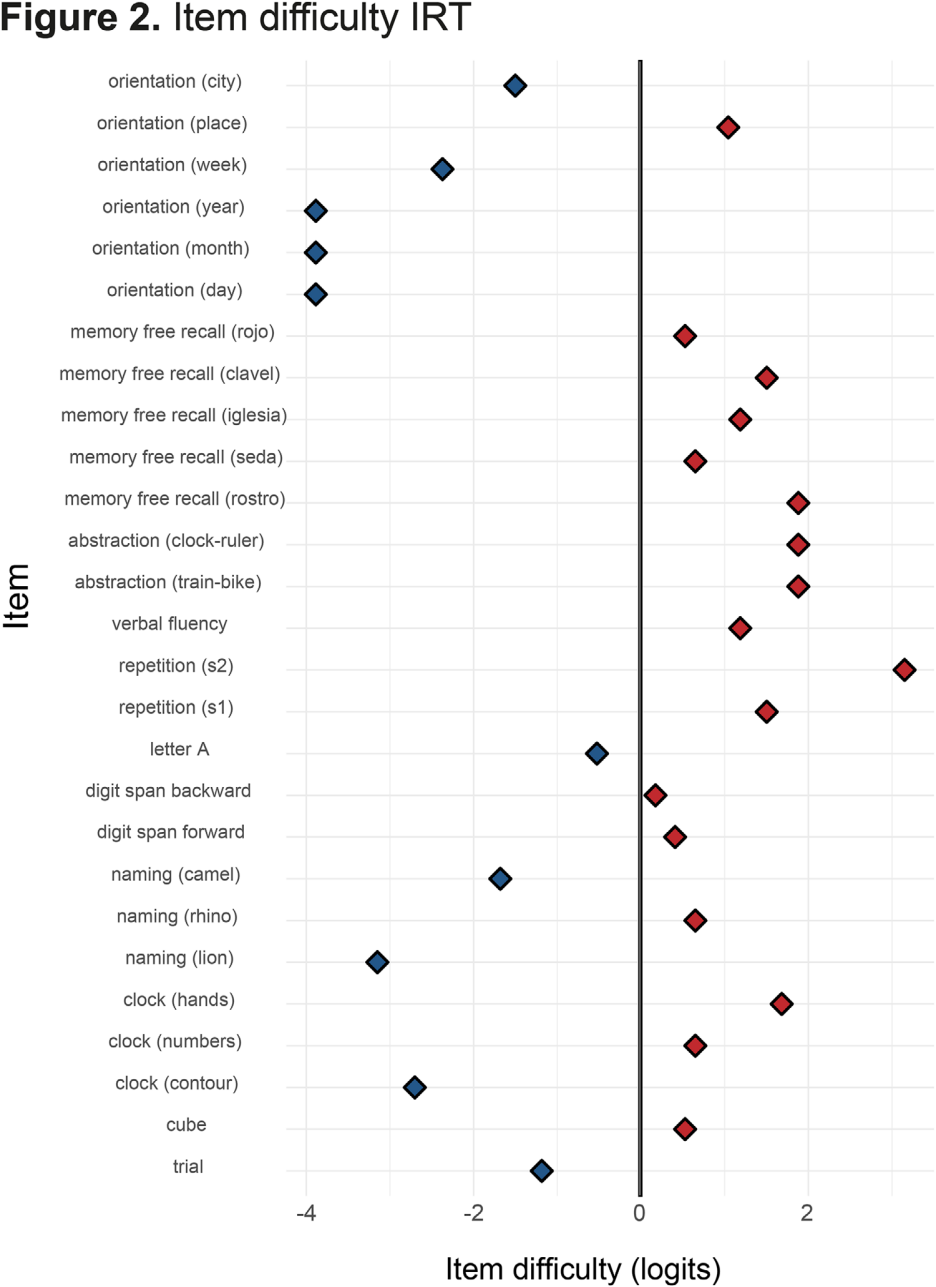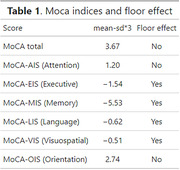# Floor‐effect in the performance of the Spanish‐language MoCA by cognitively unimpaired Peruvian individuals with low educational background

**DOI:** 10.1002/alz.090704

**Published:** 2025-01-03

**Authors:** Nilton Custodio, Ismael Luis Calandri, Rosa Montesinos, Belen Custodio, Marco Malaga, Diego Chambergo‐Michilot, Diego Bustamante‐Paytan, Katherine Aguero, Graciet Verastegui, Adrián Noriega de la Colina

**Affiliations:** ^1^ Research unit, Instituto Peruano de Neurociencias, Lima Peru; ^2^ Escuela Profesional de Medicina Humana, Universidad Privada San Juan Bautista, Lima, LIMA Peru; ^3^ Cognitive Impairment Diagnosis and Dementia Prevention Unit, Peruvian Institute of Neurosciences, Lima, Lima Peru; ^4^ Instituto Peruano de Neurociencias, Lima, Lima Peru; ^5^ Alzheimer Center Amsterdam, Amsterdam UMC, Amsterdam Netherlands; ^6^ Fleni, Buenos Aires Argentina; ^7^ Unidad de diagnóstico de deterioro cognitivo y prevención de demencia, Instituto Peruano de Neurociencias, Lima, Perú, Lima, Lima Peru; ^8^ Unidad de Investigación, Instituto Peruano de Neurociencias, Lima, Perú, Lima, Lima Peru; ^9^ Cognitive Impairment Diagnosis and Dementia Prevention Unit, Instituto Peruano de Neurociencias, Lima, Perú, Lima, Lima Peru; ^10^ Grupo de Investigación Neurociencia Efectividad Clínica y Salud Pública, Universidad Científica del Sur, Lima, Lima Peru; ^11^ Facultad de Ciencias de la Salud, Universidad Científica del Sur, Lima Peru; ^12^ Montreal Neurological Institute‐Hospital (The Neuro), Montreal, QC Canada

## Abstract

**Background:**

The Montreal Cognitive Assessment (MoCA) stands as a prominent cognitive impairment screening tool, finding widespread use globally and existing in official versions across 14 languages, including Spanish. Despite this, the challenges emerge due to the extensive variations within the Spanish language, which is not only the fourth most spoken language worldwide but also possesses significant geographic diversity, particularly evident in regions like Peru. Here, the intersection of regional nuances, low educational backgrounds, and culturally distinct tasks complicates the application of a standard MoCA version. We aimed to assess MoCA performance in individuals with both high and low education levels from an urban region of Peru to investigate the applicability of MoCA in low‐educated population.

**Methods:**

A total of 40 cognitively healthy individuals with low education (less than 6 years, mean = 3.8 ±1.8) and 40 individuals with higher education (more than 6 years, mean = 14.1 ±3.5) participated in the study. We assessed them with the Spanish version 7.0 of MoCA and estimated total score and domain indices. We fitted linear regression models to evaluate age‐ and sex‐adjusted differences between groups. We examined the presence of a floor effect in the low education group, defining it as the test’s minimum possible score surpassing the mean minus three standard deviations. This observation indicates a lack of sensitivity in capturing normal inter‐individual variation at lower values. To delve deeper, we employed a three‐parameter Item Response Theory model (IRT) to estimate the difficulty of each test item.

**Results:**

We observed significant differences in the total MoCA score (b = 9.3, 95% CI: 7.7,11, p<0.001) as well as in the memory index (MoCA‐MIS, b = 5.9, 95% CI: 4.4, 7.5, p<0.001), executive function (MoCA‐EIS, b = 5.0, 95% CI:4.0,6.0 p<0.001), attention (MoCA‐AIS, b = 5.5, 95% CI: 4.4, 6.6, p<0.001), and the remaining subscores (Figure 1). Floor effect was detected in memory, executive, language, and visuospatial indices. (Table 1) The more complex items identified were repetition and abstraction tasks (Figure 2)

**Conclusion:**

Our results shows that MoCA indices can be significantly influenced by education, floor effect is present even in healthy individuals, and certain items need to be reconsidered due to their difficulty in this context.